# Inhibitory reversal of morpheme-mediated semantic priming in L2 Chinese: embodiment conflicts in conventional action metaphor processing

**DOI:** 10.3389/fpsyg.2026.1778678

**Published:** 2026-03-31

**Authors:** Qianqian Lei, Jianqin Wang, Xingang Yang

**Affiliations:** 1 College of Chinese Language and Literature, Qufu Normal University, Qufu, Shandong, China; 2 Center for Cognitive Science of Language, Beijing Language and Culture University, Beijing, China

**Keywords:** bilingualism, conventional metaphor, embodied cognition, inhibition, L2 metaphor processing, motor simulation, second language acquisition, semantic priming

## Abstract

Semantic priming typically facilitates lexical access; however, this facilitation may reverse into inhibition under certain interferences, such as embodied conflicts in Second Language (L2) processing. For adult L2 learners, automatic literal sensorimotor simulations may disrupt metaphorical integration, potentially inducing inhibitory reversal in conventional action metaphors [e.g., Chinese “吃亏 “(chī kuī, literally “eat loss,” figuratively “suffer the loss”)]—a key gap in bilingual cognition. This study examines this reversal in L2 Chinese metaphor processing versus L1. Forty-three Vietnamese-speaking L2 learners of Chinese (HSK 5–6) and forty-seven first-language (L1) Chinese speakers completed a delayed-response semantic plausibility judgment task with morpheme-mediated semantic priming. Targets—literal, conventional metaphorical, and unrelated verb-object (VO) constructions—were each preceded by their identical verb morpheme (e.g., “吃” primes “吃亏”). Mixed-effects models revealed an opposite directional reversal in L2 learners: facilitation in literal versus unrelated baseline (shorter reaction times [RTs]; reduced errors) but inhibition in metaphorical versus unrelated baseline (elevated errors; nonsignificant RTs). In contrast to L2’s reversal pattern, L1 Chinese speakers exhibited uniform dual inhibition across literal and metaphorical conditions (elevated errors; nonsignificant RTs), with a significant Group × Condition interaction. This study reveals an L2-specific reversal of priming in action metaphors (literal facilitation vs. metaphorical inhibition, primarily evident in error rates), originating from a dynamic mismatch between embodied simulations and semantic integration, a process potentially involving increased inhibitory control demands, while remaining consistent with broader processing costs at the behavioral level. These findings offer insights into the double-edged role of L2 embodiment in language processing -- helping Literal while hurting Metaphorical, providing implications for theories of embodied cognition and bilingualism and also informing practical pedagogy in L2 acquisition.

## 1 Introduction

A core claim of psycholinguistics is that language comprehension is facilitated by preceding contextual information—a phenomenon known as semantic priming. For example, with an exposure to a prime word (e.g., “doctor”), one can typically speed up recognition or judgment of a related target (e.g., “nurse”), because associative networks are automatically activated and spread (McNamara, 2005; Meyer and Schvaneveldt, 1971; Neely, 1977, 1991).

Critically, this facilitation can be interfered with, or even reverse into inhibition. To clarify, “interference” in priming typically manifests as slowed processing or heightened effort when what the prime actually activates is the competing representations (Rohr and Wentura, 2021), but this does not alter the overall direction of the effect (e.g., reduced facilitation but still net positive priming). However, “reversal” is where an opposite effect occurs: facilitation turning into inhibition (D’Angelo et al., 2016; Tipper, 1985; Tipper and Weaver, 2008). Specifically, “inhibitory reversal” can manifest such that the main behavioral measures of priming flip from facilitation (faster responses/less errors) to inhibition (slower responses/more errors), often under conditions of strong representational conflict.

To understand inhibitory reversal would offer insights into cognitive control and conflict resolution. The present study explores this in real-time processing of conventional action metaphors (e.g., English “grasp the idea”; Chinese “吃亏” [chī kuī, suffer a loss]) by L2 learners. We propose that a specific embodied conflict, induced by a morphological priming paradigm, where the prime and target shared a morpheme (e.g., 吃chī and吃亏chī kuī), would invert priming from facilitation to inhibition in L2, signaling challenges in bilingual figurative processing.

It is well-documented that asymmetries between L1 and L2 processing are prevalent and persistent, even for L2 learners at advanced proficiency level. L2 learners, especially adult L2 learners, show their common weakness in domains like grammatical accuracy, lexical access, and pragmatic appropriateness (Bialystok, 2001; DeKeyser, 2000; Han and Odlin, 2006; Jarvis and Pavlenko, 2008; Selinker, 1972). These asymmetries are explained in dominant models of bilingual representation, such as the Revised Hierarchical Model (RHM) that attributes these asymmetries to the strength of lexical-conceptual links, positing that L2 words initially access meaning via L1 lexical mediation, with direct L2-concept connections strengthening only gradually (Kroll and Stewart, 1994; Kroll and Tokowicz, 2005; Kroll et al., 2010). Complementarily, the Shallow Structure Hypothesis (SSH) suggests L2 processing relies more on surface-level lexical and semantic cues, potentially at the expense of rapid, detailed syntactic and conceptual integration (Clahsen and Felser, 2006). The general processing delays or increased efforts in L2 are well explained in these accounts, but they are less adequate in accounting for the possible reversal on the part of L2 in online processing dynamics under specific conditions of competition, such as L1-L2 asymmetries in embodied simulation as well as abstract meaning integration.

Unlike models focusing primarily on representational strength (e.g., RHM) or processing depth (e.g., SSH), the embodied cognition framework shifts the analytical focus to “simulation” and the real-time competition it engenders. Embodied theories hold that conceptual knowledge is grounded in the human brain’s modal systems for perception, action, and emotion, and that language understanding involves re-enactment (or simulation) of these sensorimotor experiences (Barsalou, 1999, 2008, 2010; Gallese and Lakoff, 2005; Gibbs, 2012; Glenberg and Kaschak, 2002; Meteyard et al., 2012; Pulvermüller, 2005, 2013; Taylor and Zwaan, 2009; Zwaan et al., 2002; Zwaan, 2014). Embodiment is supported by the neurobiological discovery of the mirror neuron system in the premotor and parietal cortices. Mirror neurons link action perception and execution (Gallese et al., 1996), extending to action-language simulation (Rizzolatti and Arbib, 1998; Rizzolatti and Craighero, 2004). Studies consistently show action verbs (e.g., “grasp” “kick”) selectively activate motor areas via mu-rhythm desynchronization (Kemmerer, 2021; Vukovic and Shtyrov, 2014; Vukovic et al., 2017).

However, two debates within embodied cognition are directly relevant to L2 metaphor processing. One debate centers on the degree of embodiment in L2, compared to L1 (Barsalou, 1999; Glenberg and Kaschak, 2002; Vukovic and Shtyrov, 2014). While L1 acquisition is characterized by immersive, context-rich interactions that forge strong sensorimotor associations, adult L2 acquisition is often more explicit, classroom-based, and detached from rich multisensory contexts (Ellis, 2002; Tarone, 2018). Consequently, a key question is whether L2 processing engages sensorimotor simulations to the same extent and with the same automaticity as L1 processing. Converging behavioral and neurophysiological evidence suggests a profile of “reduced” or “attenuated” embodiment in L2, particularly for action language, where motor cortex engagement is often weaker or delayed compared to L1 (Bai and He, 2021; DeKeyser, 1990, 2000; Foroni, 2015; Hartshorne et al., 2018; Hayakawa and Keysar, 2018).

Another debate concerns the embodiment of abstract concepts themselves (Dove, 2011, 2020; Mahon and Caramazza, 2008; Mahon, 2015). How are concepts grounded, like “time” or “justice,” which lack direct sensorimotor referents? There are disputing claims of strong embodiment and weak embodiment. A prominent solution from Conceptual Metaphor Theory is that abstract concepts are understood via metaphorical mappings from concrete, embodied domains (e.g., TIME IS SPACE, UNDERSTANDING IS GRASPING) (Gibbs et al., 2004; Gibbs, 2016; Lakoff and Johnson, 2003, 1980). From this perspective, the comprehension of an abstract concept involves the unconscious activation of its associated metaphorical source domain, which is itself embodied (Barsalou, 1999; Gibbs, 2006; Khatin-Zadeh, 2023; Soroli, 2024). Thus, the embodiment of abstract meaning may be indirect, deriving from its metaphoric linkage to bodily experience (Stelter, 2000; Meteyard et al., 2012). Reviews, which challenge strong embodiment, argue abstracts pose generalization problems resolvable through hybrid symbolic-embodied systems (Desai et al., 2013; Thill and Twomey, 2016; Tirado et al., 2018). Cross-language priming in learners of English as a second language shows that L2 metaphor comprehension relies on L1 embodied mappings (Chen et al., 2025; Hayakawa and Keysar, 2018; Monaco et al., 2021), possibly leading to weaker or invisible embodied effects in L2 due to L1-L2 mismatches (Britz et al., 2024; Chen, 2023).

These two debates together explain why L2 metaphor processing commonly suffers general interference—delayed integration and higher error rates stemming from weaker sensorimotor links. However, under specific priming conditions—like morpheme-mediated paradigms where a literal verb strongly activates sensorimotor simulations—we argue that this interference can escalate into a qualitative reversal. This escalation arises from a core clash: L2 learners typically show certain automatic literal simulations (from L1 transfer or over-reliance on surface cues), yet weaker automatization of metaphorical mappings (due to limited cultural-linguistic exposure). When the clash occurs, the same prime that facilitates literal targets (congruent simulation) inhibits metaphorical ones (literal simulation competing with fragile figurative integration), a process being modulated by inhibitory conrol and interfered with other possible factors.

Conventional action metaphors with verb-object (VO) structures, such as English “grasp an idea” and Chinese “吃亏” (figuratively “suffer a loss”), lie at the intersection of these debates, and thus provide a revealing context for examining the clashes mentioned above. These expressions are fossilized metaphorical mappings where a concrete source domain (a physical action) provides structure for an abstract target domain (a mental state or social dynamic) (Yu, 2008). Their comprehension inherently involves a “tension” between the literal sensorimotor simulation automatically evoked by the action verb and the abstract figurative meaning that must be integrated (Feng and Zhou, 2021; Santana and De Vega, 2011). For L1 speakers, lifelong co-activation allows efficient resolution to such tensions, where the literal simulation may even facilitate metaphorical access (Littlemore et al., 2011). For L2 learners, however, resolution is fundamentally altered by the debates above. Due to reduced L2 embodiment (Debate 1), literal simulation itself may be less robust (Lu and Yang, 2025; Kühne and Gianelli, 2019), not to mention the figurative meaning of a conventional metaphor as an abstract concept. According to Debate 2, metaphors allow us to draw on concrete, familiar domains to acquire and reason about abstract concepts (Jamrozik et al., 2016). Yet, the L2 mapping is not yet fully entrenched in the conceptual system, mainly due to limited cultural-linguistic exposure, resulting in a weak embodied foundation for the figurative meaning. A specific L2 mismatch therefore occurs: an automatically activated literal simulation (e.g., of eating) clashes with a weaker metaphorical mapping to the abstract target domain (e.g., of suffering a loss). This mismatch is not merely a semantic discrepancy but a competition between different strengths and types of embodied representations.

When the embodiment clash occurs, it potentially involving increased demands on inhibitory control. We propose that when learners’ inhibitory control demands to resolve this strong clash interferes the processing, it may trigger the aforementioned qualitative reversal. Emerging evidence further highlights the role of inhibitory control as a critical mechanism in L2 figurative language processing, where metaphor interpretation involves suppressing irrelevant meanings while enhancing properties of the metaphor vehicle that are relevant for interpretation (Rubio Fernandez, 2007). This is particularly evident when additional factors, such as literal bias and L1 interference, are present. Bilinguals often bias toward literal meanings in L2 metaphors due to reduced automaticity and stronger interference from salient literal or L1-mediated activations, necessitating enhanced suppression to access figurative interpretations (Cieślicka, 2006; Heredia and Cieślicka, 2016). Behavioral and eye-tracking studies demonstrate that lower inhibitory control impairs L2 metaphor comprehension, particularly for culturally incongruent expressions, where conflicting L1 metaphorical meanings must be inhibited to resolve cross-cultural conceptual conflicts (Chen et al., 2025; George and Wiley, 2016, 2019). In reading tasks without explicit interpretation demands, inhibitory control may not significantly affect culturally congruent metaphors, yet it remains crucial for suppressing literal meanings during deeper interpretation tasks (Chen et al., 2025; Gernsbacher et al., 2001; Glucksberg et al., 2001). However, direct evidence is lacking on whether its behavioral cost is substantial enough to trigger the aforementioned qualitative reversal—especially for figurative expressions with distinct morphological compositions like Chinese VO constructions.

A typical illustration is the Chinese metaphorical VO constructure “吃香” (meaning “to be sought-after”) where L2 learners easily make errors. Learners may over-apply literal meanings or experience L1 interference, mistakenly associating it with food-related phrases such as “吃饭很香” (to eat with relish) or produce awkward abstract usages. While this primarily stems from the embodied mismatch-lacking the entrenched L1-like link between the lexical concepts and the corresponding bodily-social experience-other contributing factors may include L1-L2 semantic-conceptual mismatches (e.g., differing metaphorical extensions of the literal “吃, eat” across languages), the holistic versus decompositional processing of idiomatic expressions, or challenges in chunk-based learning of formulaic sequences. Nonetheless, these errors illustrate how automatic literal simulations, driven by the core embodied conflict and potentially involving increased inhibitory control demands, actively interfere with the integration of conventional figurative meanings in L2 processing.

Despite significant advancements in L2 metaphor research and embodied cognition, gaps remain at their intersection. Prior work has robustly revealed a general tendency that literal interpretation takes precedence over figurative interpretation (e.g., faster reading times for metaphoric expressions used literally compared to their figurative uses; Heredia and Cieślicka, 2016), typically supported by the Graded Salience Hypothesis (Giora, 2002, 2003) and the Literal Salience Model (Cieślicka, 2006; Heredia and Cieślicka, 2016) and neurocognitive studies have shown attenuated sensorimotor activity during L2 action related processing (Foroni, 2015; Birba et al., 2020) (reflecting the first debate on attenuated L2 embodiment). However, a specific prediction derived from an embodied conflict account remains insufficiently tested with direct behavioral evidence: for the same L2 learner, could the same morphological priming yield qualitatively opposite behavioral consequences (facilitation vs. inhibition) based solely on whether the target is literal or metaphorical? This “inhibitory reversal” would provide direct evidence supporting the conflicts of a strong literal simulation interfering with weaker figurative integration.

Furthermore, research specific to Chinese as a Second Language (CSL) remains limited, particularly concerning VO metaphorical constructions like “吃亏” (*chī kuī*, literally “eat loss,” figuratively “suffer the loss”) —a typical syntactically transparent but lexically unitary items (Packard, 2000). Existing studies on L2 metaphor comprehension often focus on spatial or nominal metaphors, revealing proficiency-modulated embodied effects (e.g., Wei et al., 2024; Yang and Reid, 2024). However, direct investigations of embodied conflicts, such as literal simulation interference, during the online processing of conventional VO metaphors [e.g., ‘吃亏’ (suffer a loss), ‘洗脑’ (brainwash)] are scarce. Most research highlights general processing delays or ERP differences rather than a qualitative reversal in priming dynamics. This specific gap underscores the need for a targeted paradigm to isolate literal interference within syntactically transparent but semantically semi-opaque/idiomatic VO compounds.

Therefore, this study aims to fill these interconnected gaps, by employing a morpheme-mediated semantic priming paradigm with delayed response. Specifically, it investigates whether an inhibitory reversal of priming-primarily driven by embodied conflict potentially involving inhibitory control demands, and remaining consistent with broader processing costs at the behavioral level-exists in L2 Chinese conventional action metaphor processing.

## 2 The present study

This study employs a morpheme-mediated semantic priming paradigm (e.g., using the verb “吃”/*chī*, ‘eat’ as a prime for the compound “吃亏” / *chīkuī*, ‘suffer a loss’). In this design, the prime is a free, semantically rich verb morpheme that is identical to the first constituent of the target VO compound. Crucially, presenting this morpheme in isolation strongly and automatically activates its core semantic representation, particularly its literal sensorimotor simulation (e.g., the action of eating). This paradigm is therefore suited to probe the proposed conflict between automatically activated embodied simulations and subsequent meaning integration, as it can reveal early, automatic processing differences between L1 and L2 (Gao et al., 2022). By maximizing the activation of the literal simulation prior to target presentation, it allows us to directly compare its impact across different target types. In our design, a single verb morpheme (e.g., “吃”) serves as the prime for target VO constructions that contain it. Targets are of three categories: Literal (e.g., “吃饭” / *chī fàn*, ‘eat a meal’), where the prime’s simulation aligns with the target meaning; Conventional Metaphorical (e.g., “吃亏” / *chī kuī*, ‘suffer a loss’), where the prime’s simulation conflicts with the target’s figurative meaning; and an Unrelated Control (e.g., “拿人” / *ná rén*, ‘apprehend someone’).

To specifically tap into the time window where such automatic sensorimotor simulation (see Method section for detailed explanation) and its conflict with figurative integration are hypothesized to occur, we employed a delayed-response semantic plausibility judgment task. With reaction times measured from prompt onset, this design temporally isolates the initial automatic simulation and subsequent integration processes from the immediate motor response preparation and execution, thereby allowing for more valid inferences about the underlying embodied competition.

The study included two groups of participants: Vietnamese-speaking learners of CSL at an advanced-intermediate level (HSK 5–6), and a control group of L1 Chinese speakers. There is typological similarity between Vietnamese and Chinese. Both languages are analytic and SVO, with prevalent metaphorical VO constructions (e.g., *nắm bắt* ‘grasp/catch’ meaning “comprehend,” and its VO structure *nắm bắt kiến thức* meaning “catch the knowledge”). This shared structural foundation reduces syntactic interference in the priming task, helping to isolate the effects of embodied conflict stemming from differences in metaphorical conventionalization. Moreover, all participants in the L2 group were late learners [age of acquisition (AoA) ≥ 11 years]. They gained 1 to 5 years of residential study in China after 18 years old, yet primarily within academic settings.

The embodied conflict account predicts a dissociation for L2 learners. The repeated and shared free morpheme should facilitate the processing of literal targets by pre-activating a congruent sensorimotor and semantic framework. Conversely, for metaphorical targets, the same potent literal simulation may induce interference, competing with the selection and integration of the less-embodied figurative meaning. If the L2 inhibitory control demands—which is hypothesized be less efficient at suppressing task-irrelevant, L1-like, or dominant literal activations—fails to resolve this competition swiftly, the behavioral outcome would be a reversal of the priming effect: facilitation for literals turns into inhibition for metaphors. The present study recorded and analyzed reaction times and error rates as primary dependent measures, and these two metrics may differ in their sensitivity to inhibitory costs associated with the reversal under the design.

Based on this prediction, our investigation tests three central hypotheses:

*H1*: (Facilitation for Literals): L2 learners will show significant priming facilitation for literal targets relative to unrelated baselines, reflected in shorter reaction times (RTs) and/or reduced error rates.

*H2*: (Inhibition for Metaphors): L2 learners will show significant priming inhibition for conventional metaphorical targets relative to unrelated baselines, primarily reflected in substantially elevated error rates and/or prolonged RTs.

*H3*: (L2-Specific Interaction): This dissociative pattern (literal facilitation vs. metaphorical inhibition) will be unique to the L2 group, resulting in a significant Group × Condition interaction. L1 speakers are expected to show balanced and efficient processing across conditions due to lifelong co-activation and automatized resolution of literal-figurative mappings, manifesting as comparable mild priming effects or minimal modulation in both literal and metaphorical trials, without reversal.

Behavioral data (RTs and error rates) are analyzed using linear mixed-effects (LMM) / generalized LMM (GLMM) models, accounting for variability from both participants and items. The results are discussed primarily in terms of a dynamic mismatch between embodied literal simulations and figurative integration, involving increased inhibitory control demands in resolving this embodied conflict during L2 lexical-semantic competition.

If confirmed, our hypotheses would identify the inhibitory reversal of priming as a behavioral marker of embodied conflict in L2 figurative processing. This moves beyond describing general costs to specifying the conditions that cause a qualitative change—a reversal—in a fundamental psycholinguistic process. We extend existing models (RHM, SSH, and embodied cognition accounts) by proposing a real-time simulation conflict (strong literal vs. weak figurative activation) as the core along with other possible interferes to explain this shift. The implications span theoretical accounts of the bilingual lexicon and L2 concepts, as well as practical strategies (e.g., gesture-based, immersive methods) for building stronger embodied foundations for L2 figurative competence (Jusslin et al., 2022; Reggin et al., 2023).

## 3 Materials and methods

### 3.1 Participants

A total of 92 participants were recruited from Qufu Normal University. All reported normal or corrected-to-normal vision, no history of diagnosed communicative, attentional, or behavioral disorders, and were right-handed as confirmed by the Edinburgh Handedness Inventory (Edlin et al., 2015; Oldfield, 1971).

Participants were divided into two groups. The L2 learner group consisted of 44 native Vietnamese speakers (4 male; M_age_ = 22 years, SD = 2.8, range: 18–30). They began learning Chinese formally in Vietnam at age 11 or later and had been staying in China for 1–5 years as undergraduate or postgraduate students. All L2 learners had passed the Hanyu Shuiping Kaoshi (HSK; the official Standardized Test of Chinese Proficiency) Level 5 or 6. The L1 speakers as the control group consisted of 48 L1 Chinese speakers (8 male; M_age_ = 20 years, SD = 1.9, range: 18–25). The two groups did not differ significantly in age (*p* = 0.058) or gender distribution (*p* = 0.443). All experimental procedures were conducted following the *Declaration of Helsinki*, and the experiment was approved by the Biomedical Ethics Committee of Qufu Normal University. Prior to the experiment, each participant signed the informed consent, and was compensated for their time after the experiment.

### 3.2 Experimental design and paradigm

A morpheme-mediated semantic priming paradigm was employed, using stimuli such as “吃” (chī, “eat”) as a prime for “吃亏” (chī kuī, “suffer a loss”). In this paradigm, the prime and target share an identical morpheme that is free. The experiment used a 2 (Group: L2 Chinese Learners vs. L1 Chinese Speakers, between-subjects) × 3 (Semantic Priming Relation: literal vs. metaphorical vs. unrelated control, within-subjects) mixed design. To counterbalance prime exposure in critical trials, a full set of 270 items (90 primes × 3 conditions) were counterbalanced across three Latin-square lists (each with 90 critical trials: 30 per condition), with each participant randomly assigned to one list. A representative set of experimental materials is provided in Supplementary material S1.

Of the originally recruited 92 participants (44 L2 learners; 48 L1 speakers), behavioral data from one L2 learner and one L1 speaker were excluded due to data recording failures. Thus, data from 90 participants (43 L2 learners; 47 L1 speakers) were included in the final analyses. Post-hoc sensitivity analyses were conducted using G*Power 3.1.9.7 (Faul et al., 2009) test family: repeated-measures ANOVA, within-between interaction; input parameters: repeated-measures correlation *r* = 0.5, sphericity correction *ε* = 0.75; effect size *f* (V). At *α* = 0.05 and 80% power, this 2 × 3 mixed ANOVA design could detect a minimum effect size of *f* (V) = 0.365, equivalent to Cohen’s *f* ≈ 0.183 (small-to-medium range; Cohen, 1988). This indicates sufficient sensitivity to detect subtle priming effects with the final sample. Note that this analyses approximates the traditional repeated-measures ANOVA framework, whereas actual analyses used more flexible linear mixed-effects models. By incorporating item random effects and random slopes for individual differences, LMMs typically yield more conservative (i.e., smaller) effect size estimates than ANOVA, thereby enhancing inferential robustness (Brysbaert and Stevens, 2018).

### 3.3 Stimuli

Primes were single-syllable action verbs (e.g., “洗”/xǐ, ‘wash’; “砍”/kǎn, ‘chop’; see Table 1). Targets were disyllabic VO constructions, classified into three semantic priming relations (see Table 1):

(a) Literal: Transparent compositional meanings directly derived from the verb’s literal action (e.g., “洗衣”/xǐ yī, ‘wash clothes’).
(b) Conventional Metaphorical: Non-compositional, figurative meanings extended from verb’s bodily actions via conceptual metaphor (e.g., “洗脑”/xǐ nǎo, literally ‘wash brain’, meaning ‘brainwash’; 砍价/kǎn jià, literally ‘chop price’, metaphorical meaning ‘bargain’). These were operationally defined as fixed collocations whose meanings could not be directly computed from their literal components, rated by experts using a 7-point Likert scale on figurative distance, which ensured the figurative opacity.
(c) Unrelated: Semantically plausible but shared no semantic relationship with the prime action verb (e.g., “拿人”/ ná rén, ‘apprehend someone”), serving as a baseline without the confound of verb morpheme repetition.

**Table 1:** Examples of primes and targets across conditions.

Semantic priming relation	Prime	Target
Literal	洗 xǐ (wash)	洗衣 xǐ yī (washing clothes)
Conventional metaphorical	洗 xǐ (wash)	洗脑 xǐ nǎo (brainwash literally ‘wash brain’)
Unrelated	洗 xǐ (wash)	拿人 ná rén (to apprehend someone)

Pinyin and English translations for non-Chinese readers.

Targets across the three conditions were matched on linguistic features: stroke count (from *Xinhua Dictionary*), frequency (from CCL corpus, 2024 version, 4.75 billion characters; estimated words ~2.97 billion, 1.6 characters/word ratio; Zhan et al., 2003, 2019). Subjective ratings using 7-point Likert scales on four features (familiarity, semantic transparency, concreteness, action-relatedness) were collected from 32 native raters, and figurative distance from 7 linguists. Semantic transparency was defined as the degree to which each morpheme’s meaning relates to the whole word (Tse et al., 2016), and this study employs a whole-word semantic transparency rating method. Figurative distance quantified the conceptual separation from the verb’s literal action to its abstract metaphorical extension, rated by 7 linguists on a 7-point scale [1 = very close to literal action (low distance, e.g., transparent collocations), 7 = very distant from literal action (high distance, e.g., opaque idioms); *ICC* = 0.954]. This ensured standardized figurative opacity across metaphorical targets, with higher scores indicating greater abstraction and potential embodied conflict.

Descriptive statistics (see Table 2) and subsequent inferential tests confirmed no significant differences in baseline features (e.g., stroke count, frequency, familiarity, all *ps* > 0.05) but confirmed expected divergences in semantic properties: metaphorical targets exhibited lower transparency, concreteness, action-relatedness, along with higher figurative distance, compared to literal/unrelated conditions (all Dunn post-hoc *ps* < 0.001). These patterns affirm the conditions’ distinctiveness while ensuring baseline matching.

**Table 2:** Descriptive statistics for target word features across semantic conditions.

Feature	Literal (M/SD)	Metaphorical (M/SD)	Unrelated (M/SD)
Stroke count	15.84 (3.84)	15.73 (3.70)	15.97 (3.20)
Log CPM frequency	0.99 (0.87)	0.91 (0.74)	1.04 (0.82)
Familiarity	6.30 (0.18)	6.28 (0.19)	6.33 (0.19)
Usage frequency	5.10 (1.06)	4.69 (1.20)	5.03 (1.05)
Semantic transparency	6.06 (0.46)	4.34 (0.66)	5.94 (0.72)
Concreteness	5.74 (0.68)	3.71 (0.64)	5.52 (0.94)
Action-relatedness	4.47 (0.81)	3.11 (0.47)	4.14 (0.87)
Figurative distance	1.50 (0.64)	4.76 (0.75)	1.79 (1.04)

*N* = 270 (90 × 3condition); CPM = counts per million; M = mean, SD = standard deviation.

Additionally, 20 independent L2 learners of Chinese (HSK Levels 5–6, matched to the experimental group on demographics and proficiency) rated the targets for semantic plausibility on a 7-point scale (1 = highly implausible, 7 = highly plausible). Results confirmed high plausibility across conditions (M_overall_ = 6.12, SD_overall_ = 0.89), with no significant differences (*p* = 0.62): literal (*M* = 6.28, SD = 0.72), metaphorical (*M* = 6.05, SD = 0.95), unrelated (*M* = 6.03, SD = 0.98). All exceeded the task threshold of 5.0, indicating above-chance endorsement per L2 norming conventions. This demonstrates that, despite lower semantic transparency, L2 learners reliably endorsed Metaphorical targets as plausible.

### 3.4 Procedure

The experiment was conducted at Qufu Normal University. Participants were seated approximately 60 cm from a 19.5-inch Dell LCD monitor (1,440 × 900 resolution, 60 Hz refresh rate). The stimuli were presented using E-Prime 3.0 software.

Participants performed a visual lexical semantic plausibility judgment task: They passively read prime-target pairs, then judged the target’s semantic plausibility by pressing two keys (‘j’ key = plausible; ‘f’ key = implausible). Instructions were displayed on the screen; participants pressed the key “Enter” to proceed after having understood the task requirements.

**Figure 1:**
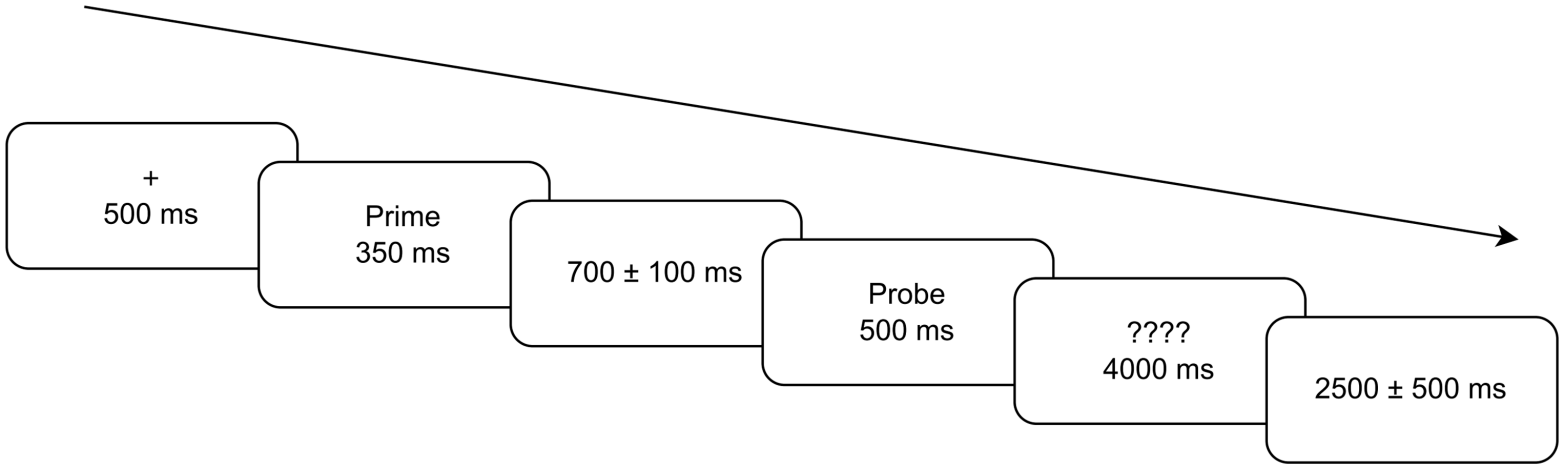
Time course of a single experimental trial.

Each trial proceeded as follows (see Figure 1): A centrally presented fixation cross ‘+’ appeared for 500 ms, followed by the prime for 350 ms (displayed in 50 pt. Heiti font against a white background). After an ISI of 700 ± 100 ms (jittered in 10 ms steps to minimize anticipation), the target appeared for 500 ms (also displayed in 50 pt. Heiti font against a white background), with a prohibition of key-presses during this 500 ms target window. RTs were measured from prompt onset. The response prompt (‘????’) appeared immediately following target offset, with no visual overlap. The prompt remained for 4,000 ms, prompting participants to make rapid, accurate plausibility judgments on the target during this latency window. Once participants pressed a button, a blank screen would be triggered; alternatively, timeouts (no response) would advance automatically after 4,000 ms, and the trials with no response were rejected. A jittered ITI blank screen (2,500 ± 500 ms, jittered in 10-ms steps) separated trials.

A delayed-response paradigm was employed to temporally dissociate the initial automatic sensorimotor simulation (target presentation window) from later motor response preparation and execution (button pressing during the response window):

(1) 500 ms time window for target presentation. On the one hand, the target remained on screen for 500 ms-well within the time window in which action semantics typically elicit sensorimotor simulation [approximately 150–600 ms post-onset, as indexed by mu-rhythm desynchronization (ERD)] (Hauk et al., 2008; Pulvermuller, 1999; Vukovic and Shtyrov, 2014). On the other hand, motor responses were prohibited, because preparing or executing a button press during target presentation can itself activate sensorimotor cortices and confound pure simulation processes (action words and motor actions share common cortical representations; Boulenger et al., 2006). Thus, this time window allows for participants’ early semantic processing as well as automatic sensorimotor simulation while minimizing contamination from motor preparation artifacts, thereby ensuring the underlying embodied competition between literal simulation and figurative integration.
(2) 4,000 ms time window for response. Once participants pressed a button, a blank screen was triggered. Though a maximum of 4,000 ms was permitted, participants were explicitly instructed to make judgments as quickly and accurately as possible once the prompt appeared. As confirmed in RT data, distribution checks showed no evidence of strategic slowing; in data processing (see Section 3.5.1), strict extremes/outliers exclusion criteria were employed.

Participants completed a total of 150 trials: 90 critical (30 per condition) and 60 fillers. Filler trials (*n* = 60 per participant, identical across lists, 95% implausible VO constructions) comprised two-thirds the number of critical trials to maintain a ~ 70:30 plausibility ratio. All trials were randomly presented on the screen, with a full session (~16 min) comprising 3 blocks (~3–4 min each; 50 trials/block). Between blocks, participants were permitted to take a break, and resumed via any keypress.

Prior to formal trials, participants engaged in 8 practice trials (balanced across conditions, no overlap with experimentals) with accuracy feedback. Up to three iterations were allowed until ≥ 90% accuracy; the experimenter verbally confirmed task mastery.

### 3.5 Data analysis

#### 3.5.1 Data processing

Behavioral data from the semantic plausibility judgment task were analyzed, with reaction time (RT) and accuracy (ACC) as the primary outcome measures.

For RT analysis, only correct trials (ACC = 1) were retained (81.81% of total, *N* = 6,627; 93.26% for L1, 69.30% for L2). Extreme/ Outlier RTs were further excluded: (1) fixed absolute cutoffs as extremes: RTs ≤ 150 ms (anticipatory responses) or RTs ≥ 1,500 ms (inattentional lapses or external distractions); followed by (2) outlier trials exceeding ±2.5 *SD* from each participant’s mean RT within each condition. To demonstrate the robustness of this extremes/outliers criteria, we conducted a sensitivity analysis in *R* (v4.2.3; R Core Team, 2023), testing 24 different combinations of criteria (lower cutoffs: 80/100/150/200 ms; upper cutoffs: 1000/1500/2000 ms; SD multipliers: 2.5/3.0). The main effects of Group and Condition, as well as the critical Group × Condition interaction, remained highly significant across all 24 criteria, with highly consistent descriptive patterns. We report results based on the conservative, common, and strict criterion (lower = 150 ms or upper = 1,500 ms, ± 2.5 SD) as the primary analysis, which also demonstrates superior performance across multiple model diagnostic dimensions. This cleaning procedure removed 10.16% of correct trials (12.42% for L2 learners, 8.62% for L1s), yielding 5,954 valid trials for the final RT analysis (73.51% of the original total; 85.22% for L1, 60.70% for L2 learners). To meet approximate normal distribution, RTs were natural log-transformed (logRT) prior to statistical modeling.

For ACC (binary: 1 = correct, 0 = incorrect) analysis, all 8,100 trials were included and analyzed as error rates (Error = 1 – ACC). Descriptive statistics (means, *SD*s, and trial counts) for ACC and RT were computed separately for each Group and Condition using the *dplyr* package (v2.0.0; Wickham et al., 2019).

#### 3.5.2 Statistical analysis

All statistical analyses were conducted in *R* (v4.2.3; R Core Team, 2023). Mixed-effects models were fitted via the *afex* package (v1.5.0; Singmann et al., 2023), which interfaces with *lme4* (v1.1.37; Bates et al., 2015). The LMM models for logRT used Type III sums of squares with Satterthwaite degrees of freedom, while the GLMM models (binomial logit) for error rates used likelihood ratio tests (LRT).

The fixed-effects structure for both models was Group × Condition, testing the main effects of participant group (L2 learners vs. L1 speakers) and semantic priming condition (literal vs. conventionally metaphorical vs. unrelated), and their interaction.

The random-effect structure was determined using a data-driven, step-up approach (Matuschek et al., 2017). We began with a baseline model containing random intercepts for participants (Subject) and target word items (Item). We then incrementally tested more complex random-effect structures. A model that added by-subject random slopes for Condition (i.e., (1 + Condition | Subject) + (1 | Item)) successfully converged. In contrast, models attempting to include by-item random slopes or other, more complex structures either failed to converge or produced singular fit warnings and were therefore not employed further. Likelihood ratio tests confirmed that the model with by-subject slopes provided a significantly better fit than the intercept-only baseline for both RTs [*χ*^2^ (5) = 41.87, *p* < 0.001; *Δ*AIC = −34] and error rates [*χ*^2^ (5) = 169.56, *p* < 0.001; *Δ*AIC = −160]. Consequently, this maximal converging structure was retained. The final models were specified as:


$$\begin{array}{l} \mathrm{RT}\;(\mathrm{LMM}):\text{logRT}\sim \text{Group}\times \text{Condition}\\ +(1+\text{Condition}\;|\;\text{Subject})+(1\;|\;\text{Item}) \end{array}$$

$$\begin{array}{l} \text{Error}\;(\text{GLMM}):\text{Error}\sim \text{Group}\times \text{Condition}\\ +(1+\text{Condition}\;|\;\text{Subject})+(1\;|\;\text{Item}) \end{array}$$

For the LMM, significance of fixed effects was assessed using Satterthwaite’s approximation for degrees of freedom.

For the GLMM, significance was determined via likelihood ratio tests (LRTs). Model fit was quantified using marginal *R*^2^ and conditional *R*^2^ (*performance* package, v0.15.1; Lüdecke et al., 2021).

Categorical predictors (Group and Semantic Condition) was analyzed using sum-to-zero effect coding (via contr.sum() in R through the default setting of afex:mixed() with check.contrasts = TRUE). Specifically, L1 speakers of Group and the unrelated condition of Semantic Condition were separately set as the respective base levels for matrix construction (see Supplementary Table S1). Importantly, under sum-to-zero coding there is no traditional reference level compared with treatment coding: the model intercept represents the grand mean across all levels, and each coefficient represents that level’s deviation from the grand mean. For the three-level Semantic Condition factor, this resulted in two orthogonal contrasts: Contrast 1 comparing the unrelated condition to the average of the metaphorical and literal conditions, and Contrast 2 directly comparing the metaphorical versus literal conditions (the unrelated condition received a weight of 0 in Contrast 2). Exact contrast matrices are provided in Supplementary Table S1.

Effect sizes are reported as partial omega-squared (*ω*^2^) with 95% confidence intervals (CIs) for the LMM (*effectsize* package, v1.0.1; Ben-Shachar et al., 2020), and as odds ratios (OR) with 95% CIs for the GLMM.

Additionally, for the GLMM we computed semi-partial *R*^2^ values using the *r2beta* function from the *r2glmm* package (v0.1.3; Jaeger, 2022), which quantifies the proportion of variance uniquely attributable to each fixed effect.

In the presence of significant interactions, planned simple effects were conducted (comparing Conditions within each Group and Groups within each Condition) using pairwise contrasts via *emmeans* package (v1.11.2.8; Lenth, 2024), with *p*-values adjusted using Tukey honest significant difference (HSD) for family-wise error control. For these *post-hoc* analysis, *emmeans* utilized treatment contrasts (contr.treatment) by default, with the reference levels set as L1 speakers for Group and the unrelated condition for Condition.

Model diagnostics were performed to ensure validity. Multicollinearity was low for all predictors (all VIFs < 1.4; *car* package, v3.1–1; Fox and Weisberg, 2019). Residual diagnostics were conducted using the *DHARMa* package (v0.4.7; Hartig, 2024).

For the GLMM, simulated residuals showed no significant deviation from uniformity (Kolmogorov–Smirnov test, KS, *p* = 0.76), no overdispersion (*p* = 0.98), and an acceptable outliers (*p* = 0.80).

For the LMM, DHARMa diagnostics indicated no overdispersion (*p* = 0.71), the outlier rate was low (0.72%, *p* = 0.56), but the KS test was significant (*p* < 0.001). Actually, it’s a common outcome with large sample sizes (*N* = 6,359), and visual inspection of diagnostic plots suggested no severe violations.

To verify the robustness of the parameter estimates, particularly in light of residual non-normality in the LMM and the high correlations among random slopes in both models (*r* = −0.97 for the GLMM and *r* = −0.73 for the LMM), nonparametric bootstrapping (1,000 resamples) was performed using the *bootMer* function in *lme4* (v1.1.37). The resulting 95% percentile CIs for both models overlapped substantially with the standard Wald intervals (LMM, |Bias| ≤ 0.0014 logRT units; GLMM, |Bias| ≤ 0.008 logit units), confirming the stability of the estimates.

## 4 Results

### 4.1 Descriptive statistics

**Figure 2:**
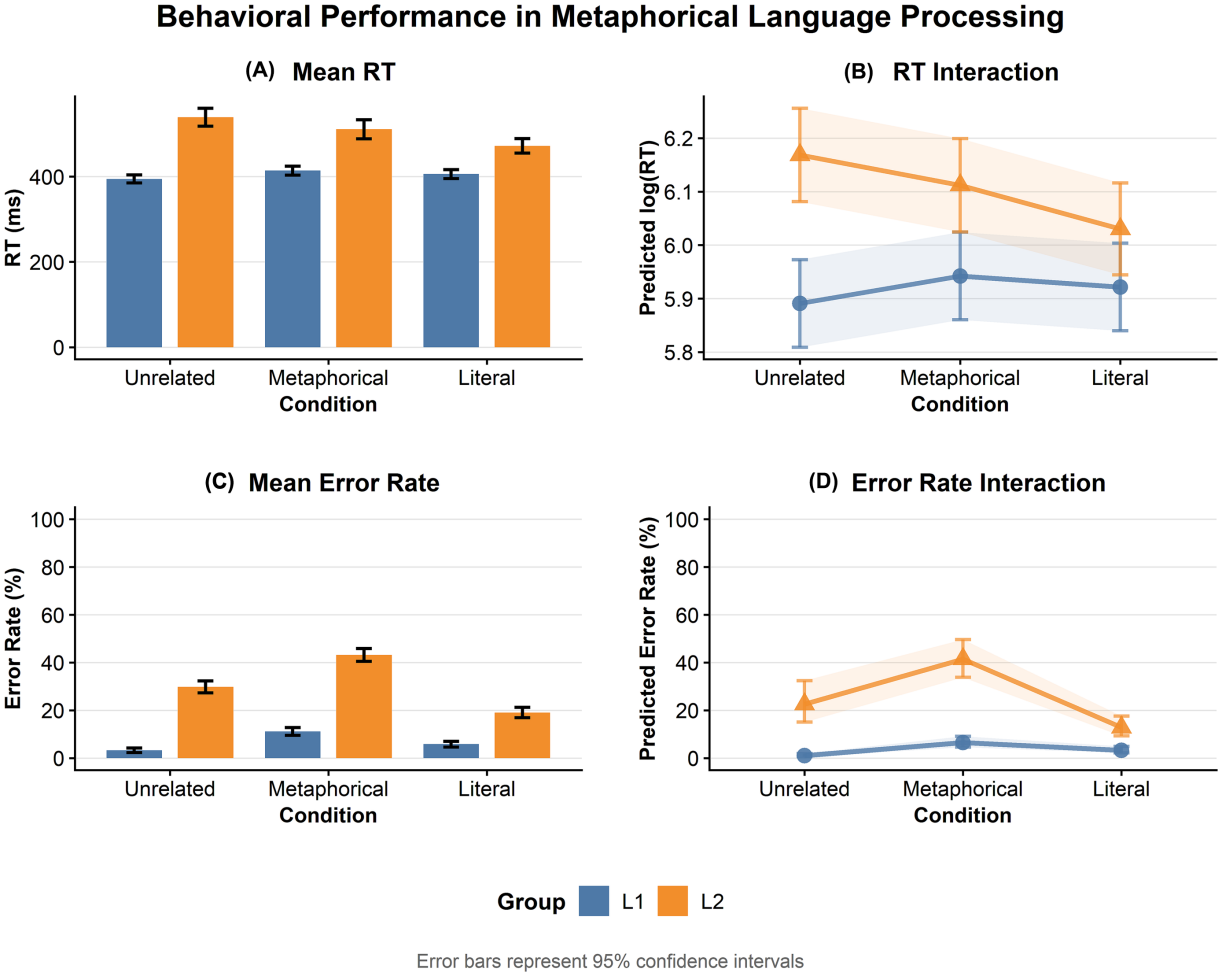
Behavioral performance in figurative language processing among first language (L1) of Chinese speakers and second language (L2) learners of Chinese. **(A)** Mean RTs (ms) and **(C)** mean error rates (%) by group and condition (Unrelated, Metaphorical, Literal). Lower error rates indicate higher accuracy. Error bars represent 95% confidence intervals (CIs). **(B,D)** Model-predicted values showing Group × Condition interactions (linear mixed-effects model for RT; binomial generalized linear mixed-effects model for error rate). Shaded areas and error bars indicate 95% CIs of marginal means. Data in **(A)** and **(C)** are raw means ± 95% CI; data in **(B)** and **(D)** are model-based estimated marginal means ± 95% CI. Due to near-ceiling accuracy in the L1 group (Metaphorical and Literal conditions), CIs in panel **(D)** for this group are with lower bounds approaching 0%.

Descriptive statistics for RTs and error rates are presented in Table 3 and visualized in Figure 2. For RT analyses, only correct trials were included after removing extreme values/ outliers (see Method section).

**Table 3:** Descriptive statistics for error rates and reaction times (RT s) by group and condition.

Group	Condition	*n_1_* (Total trials)	Error rate (M ± SD)	*n_2_* (Valid RT trials)	RT (ms; M ± SD)
L1 speakers	Unrelated	1,410	0.03 ± 0.18	1,252	394 ± 174
	Metaphorical	1,410	0.11 ± 0.31	1,150	414 ± 182
	Literal	1,410	0.06 ± 0.23	1,203	406 ± 189
	Overall	4,230	0.07 ± 0.25	3,605	404 ± 181
L2 Learners	Unrelated	1,290	0.30 ± 0.46	796	539 ± 305
	Metaphorical	1,290	0.43 ± 0.50	640	511 ± 289
	Literal	1,290	0.19 ± 0.39	913	472 ± 260
	Overall	3,870	0.31 ± 0.46	2,349	505 ± 285
Overall	Unrelated	2,700	0.16 ± 0.37	2,048	451 ± 244
	Metaphorical	2,700	0.25 ± 0.44	1,790	449 ± 231
	Literal	2,700	0.12 ± 0.33	2,116	434 ± 224
	Overall	8,100	0.18 ± 0.39	5,954	444 ± 233

n_1_ = total trials for error rates; n_2_ = number of trials after RT cleaning (correct trials excluding extremes/outliers: RT≤150 ms or RT ≥1,500 ms; ±2.5 SD trimming on a per-participant and per-condition basis); RTs are untransformed means from cleaned data. Overall rows aggregate across conditions within groups or across all levels. Data reflect slight imbalance in total trials per cell (e.g., due to unequal group sizes and cleaning of RT trials).

Overall, L1 speakers responded substantially faster (*M* = 404 ms) than L2 learners (*M* = 505 ms). Within the L2 learner group, mean RTs were shortest for literal targets (*M* = 472 ms, SD = 260), intermediate for metaphorical targets (*M* = 511 ms, SD = 289), and longest for unrelated targets (*M* = 539 ms, SD = 305), a pattern indicating literal facilitation. In contrast, L1 speakers’ RTs were relatively comparable across conditions (unrelated: *M* = 394 ms, SD = 174; literal: *M* = 406 ms, SD = 189; metaphorical: *M* = 414 ms, *SD = 182*), with no clear advantage for any target type.

Error rates were generally low for L1 speakers (overall *M* = 7%) but markedly elevated for L2 learners (overall *M* = 31%). Error rates were highest for metaphorical targets in both groups (L1: 11% ± 0.31; L2: 43% ± 0.50), with L2 learners showing a particularly pronounced cost compared to their literal (19%) and unrelated (30%) baselines. Consequently, group differences in accuracy were most dramatic for metaphorical primes and smallest—though still present—for literal primes.

### 4.2 Linear mixed-effects model for log-transformed RTs

The LMM revealed a significant main effect of Group, *F* (1, 87.59) = 10.60, *p* = 0.002, *ω*_*p*^2^ = 0.10 [95% CI: 0.02, 1.00].^1^ L1 speakers responded significantly faster overall compared with grand mean (see Table 4 for fixed effects), The estimated marginal means confirmed this pattern (*M*_L1_ = 5.92 log ms vs. *M*_L2_ = 6.10 log ms). There was also a significant main effect of Condition, *F* (2, 92.04) = 7.98, *p* < 0.001, *ω*_*p*^2^ = 0.13 [95% CI: 0.03, 1.00].

**Table 4:** Fixed effects from the linear mixed model of log-transformed RT.

Predictor	Estimate (*b*)	*SE*	df	*t* value	*p* value	95% CI (Wald)	95% CI (Bootstrap)
(Intercept) (Grand Mean)	6.011	0.029	88.71	210.45	< 0.001	[5.9550, 6.0669]	[5.9554, 6.0693]
Group Contrast1 (L1 vs. Grand Mean)	−0.093	0.028	87.59	−3.26	0.002	[−0.148, −0.037]	[−0.1483, −0.0405]
Condition Contrast 1	0.019	0.010	85.60	1.79	0.076	[−0.0017, 0.0394]	[−0.0027, 0.0376]
Condition Contrast 2	0.016	0.009	99.46	1.69	0.095	[−0.0026, 0.0350]	[−0.0029, 0.0367]
Group Contrast 1 × Condition Contrast1	−0.046	0.010	74.79	−4.62	< 0.001	[−0.0658, −0.0266]	[−0.0663, −0.0266]
Group Contrast 1 × Condition Contrast 2	0.008	0.009	87.32	0.86	0.391	[−0.0099, 0.0256]	[−0.0090, 0.0264]

b = unstandardized coefficients on logRT scale (positive = slower RT relative to grand mean). Sum-to-zero contrasts was used when fitting the LMM model (contr.sum via afex). L1 speakers of Group and the unrelated condition of Condition are set as the respective base levels for matrix construction (see Supplementary Table S1). Coefficients represent deviations from the grand mean. The model formula is logRT ~ Group * Condition + (1 + Condition | Subject) + (1 | Item). df from Satterthwaite approximation. p-values from t-tests (Satterthwaite df); ANOVA (Type III, S-method): Group *F* (1, 87.59) = 10.60, *p* = 0.002; Condition *F* (2, 9,204) = 7.98, *p* < 0.001; Interaction F(2, 81.36) = 14.27, *p* < 0.001. 95% CIs for estimates are Wald-based; Bootstrap CIs (percentile-based; 1,000 bootMer resamples, nsim = 1,000, seed = 123; via lme4), closely overlap with Wald CIs (all |bias| ≤ 0.0014 logRT units), confirming stability. Model fit: Marginal *R*^2^ = 0.047, Conditional *R*^2^ = 0.421 (Nakagawa and Schielzeth method via performance package). VIFs < 1.4 (car package). Data: N = 5,954 trials (90 subjects, 268 items). Interpretation of contrasts: *Group Contrast 1 (b = −0.093): deviation of L1 speakers from the grand mean (L2 deviation = +0.093; full L2 − L1 difference = 0.186 logRT units, ≈ 20.4% slower for L2 learners). *Condition Contrast 1 (b = 0.019): unrelated vs. average of metaphorical and literal. *Condition Contrast 2 (b = 0.016): metaphorical vs. literal.

Critically, the Group × Condition interaction was significant, *F* (2, 81.36) = 14.27, *p* < 0.001, *ω*_p^2^ = 0.24 [95% CI: 0.11, 1.00] (see Figure 2), indicating that the effect of semantic priming differed markedly between L2 learners and L1 Chinese speakers. Follow-up simple effects analyses (see Table 5 for full details) clarified this interaction. For L2 learners, literal targets produced significantly faster RTs than unrelated targets (*b* = −0.138, *p* < 0.001); in contrast, metaphorical targets did not differ significantly from unrelated targets (*b* = −0.057, *p* = 0.095), although they were significantly slower than literal targets (*b* = 0.082, *p* = 0.001). For L1 speakers, no significant RT differences were shown across any conditions: metaphorical vs. unrelated (*b* = 0.051, *p* = 0.070), literal vs. unrelated (*b* = 0.03, *p* = 0.332), and metaphorical vs. literal (*b* = 0.021, *p* = 0.523). Thus, the interaction arose because the same prime facilitated literal processing but produced no facilitation for metaphorical processing only in L2 learners.

**Table 5:** Simple effects analyses from the LMM of log-transformed RT.

Analysis	Contrast	Estimate (*b*)	SE	df	*t* ratio	*p* value (Tukey)	95% CI
Within L1	Metaphorical–unrelated	0.051	0.027	85.7	2.11	0.070	[−0.008, 0.122]
	Literal–unrelated	0.031	0.025	91.3	1.46	0.333	[−0.023, 0.096]
	Metaphorical–literal	0.021	0.022	88.9	0.94	0.523	[−0.032, 0.073]
Within L2	Metaphorical–unrelated	−0.057	0.032	120.2	−2.23	0.095	[−0.145, 0.005]
	Literal–Unrelated	−0.138	0.028	111.8	−5.74	< 0.001	[−0.230, −0.095]
	Metaphorical–literal	0.082	0.026	129.8	3.62	0.001	[0.032, 0.153]
Between Groups (L2 – L1)	In unrelated condition	0.278	0.071	88.2	4.64	< 0.001	[0.187, 0.467]
	In metaphorical condition	0.170	0.071	88.7	2.82	0.006	[0.059, 0.340]
	In literal condition	0.109	0.071	88.0	1.79	0.071	[−0.014, 0.270]

b = unstandardized differences on logRT scale (positive b = longer RT for second level). Tukey HSD-adjusted for family-wise error control. Degrees-of-freedom from Kenward-Roger. Post-hoc pairwise contrasts via emmeans apply treatment coding. Data: N = 5,954 trials (90 subjects, 268 items).

The LMM model showed good fit (marginal *R*^2^ = 0.047, conditional *R*^2^ = 0.421), with random effects analysis indicating substantial between-subject variability in baseline logRT (intercept SD = 0.27) and a strong negative correlation between the two Condition Contrast slopes (*r* = −0.73), indicating a trade-off in individual sensitivity to the priming relations.

### 4.3 Generalized linear mixed-effects model for error rates

The GLMM revealed a significant main effect of Group, *χ*^2^ (1) = 100.90, *p* < 0.001. As shown in Table 6 for fixed effects, L1 speakers showed significantly lower errors overall compared with grand mean. Estimated marginal means confirming this pattern (error rate: L1 = 0.03%; L2 = 0.23%). There was also a main effect of Condition, *χ^2^* (2) = 42.45, *p* < 0.001. As shown in Table 6, both Condition Contrast 1 and Condition Contrast 2 are significant in errors. Main effect of Condition was significant, *χ^2^* (2) = 42.45, *p* < 0.001. Condition Contrast 1 is significant in errors (*β* = −0.55, OR = 0.58, *p* < 0.001), and Condition Contrast 2 is also significant (*β* = 0.85, OR = 2.34, *p* < 0.001; see Table 6).

**Table 6:** Fixed effects from the generalized linear mixed model of error rates.

Predictor	Estimate (log odds)	*SE*	*z* value	*p* value	OR	95% CI (Wald OR)	95% CI (Bootstrap OR)
Intercept (Grand Mean)	−2.35	0.12	−19.81	<0.001	0.10	[0.08, 0.12]	[0.07, 0.15]
Group Contrast 1 (L1 vs. Grand Mean)	−1.19	0.09	−12.73	<0.001	0.30	[0.25, 0.37]	[0.25, 0.37]
Condition Contrast 1	−0.55	0.16	−3.42	<0.001	0.58	[0.42, 0.79]	[0.42, 0.79]
Condition Contrast 2	0.85	0.13	6.63	<0.001	2.34	[1.82, 3.00]	[1.82, 3.00]
Group Contrast 1 × Condition Contrast 1	−0.48	0.13	−3.84	<0.001	0.62	[0.48, 0.79]	[0.48, 0.79]
Group Contrast 1 × Condition Contrast 2	0.03	0.08	0.35	0.72	1.03	[0.88, 1.21]	[0.88, 1.21]

Effect coding (contr.sum via afex), with L1 speakers and the unrelated condition set as the respective base levels for matrix construction (see Supplementary Table S1). Coefficients represent deviation from grand mean on logit scale (positive *β* = higher log-odds of error). OR = exp(β); 95% CIs for OR were back-transformed from logit scale: Wald asymptotic (used in model fitting/analysis); Bootstrap (percentile method; via bootMer, 1,000 resamples; |bias| ≤ 0.008). Model: binomial logit (Error ~ Group * Condition + (1 + Condition | Subject) + (1 | Item)); ANOVA (Type III, LRT): Group *χ*^2^(1) = 100.90, *p* < 0.001; Condition *χ*^2^(2) = 42.45, *p* < 0.001; Interaction *χ*^2^(2) = 29.99, *p* < 0.001. Data: N = 8,100 trials (90 subjects, 268 items). *Group Contrast 1 (b = −1.19): deviation of L1 speakers from grand mean (L2 deviation = +1.19; full L2 − L1 difference = 2.38 log-odds units, OR ≈ 10.8). *Condition Contrast 1 (b = −0.55): unrelated vs. average of metaphorical and literal. *Condition Contrast 2 (b = 0.85): metaphorical vs. literal.

The Group × Condition interaction was again significant, *χ^2^* (2) = 29.99, *p* < 0.001 (see Figure 2). Follow-up simple effects analyses (see Table 7) clarified the interaction showed that L2 learners made significantly more errors than L1 speakers in all conditions (all ps < 0.001), with the largest gap in metaphorical targets. Within L2 learners, error rates were highest for metaphorical targets, significantly higher that both unrelated (OR = 2.43, *p* = 0.006) and literal targets (OR = 4.80, *p* < 0.001), while literal targets produced fewer errors than unrelated targets (OR = 0.51, *p* = 0.043). In L1 speakers, metaphorical targets also elicited the highest error rates (exceeding unrelated OR = 6.76, *p* < 0.001 and literal OR = 2.06, *p* = 0.007), but the disparity between metaphorical and literal conditions was more than twice as large in L2 (OR = 4.80) than in L1 (OR = 2.06). L1 speakers made more errors in literal than unrelated targets (OR = 3.2, *p* = 0.003).

**Table 7:** Simple effects analyses from the GLMM of error rates.

Analysis	Contrast	OR (estimate)	*SE*	*z* value	*p* (Tukey)	95% CI
Within L1	Metaphorical – Unrelated	6.76	2.30	5.30	< 0.001	[2.90, 15.67]
	Literal – Unrelated	3.27	1.18	3.28	0.003	[1.40, 7.62]
	Metaphorical – Literal	2.06	0.50	3.01	0.007	[1.17, 3.62]
Within L2	Metaphorical – Unrelated	2.43	0.70	3.08	0.006	[1.24, 4.77]
	Literal – Unrelated	0.51	0.56	−2.40	0.043	[0.26, 0.98]
	Metaphorical – Literal	4.80	0.99	7.64	< 0.001	[2.97, 7.78]
Between groups (L2 vs. L1)	In Unrelated Condition	28.25	10.47	8.99	< 0.001	[13.64, 58.48]
	In Metaphorical Condition	10.20	1.84	12.56	< 0.001	[7.08, 14.62]
	In Literal Condition	4.37	0.95	6.82	< 0.001	[2.86, 6.68]

OR = odds ratios (>1 = higher errors for first level). Tukey HSD-adjusted for family-wise error control. CIs from confint on contrasts (Tukey HSD level). z from likelihood ratio tests. Post-hoc pairwise contrasts via emmeans apply treatment coding, while model fitting used effect coding (contr.sum). Data: N = 8,100 trials (90 subjects, 268 items).

The GLMM model provided good fit (marginal *R*^2^ = 0.262, conditional *R*^2^ = 0.553), with notable trade-offs in random slopes (e.g., r = −0.97 between Condition Contrasts), highlighting individual differences in sensitivity to the priming manipulation.

## 5 Discussion

### 5.1 Summary of findings and hypothesis recap

In the present study, we investigated the dynamics of semantic priming in the real-time processing of conventional action metaphors among intermediate-advanced Vietnamese-speaking learners of CSL (HSK Levels 5–6) compared to the L1 Chinese speakers. We employed a morpheme-mediated semantic priming paradigm with delayed-response semantic plausibility judgments. Grounded in embodied cognition and bilingual processing researches, we hypothesized that automatic literal sensorimotor simulations would facilitate literal trials (H1) but elicit inhibitory reversal in metaphorical trials (H2), with L2-specific interactions reflecting developmental embodied conflict (H3). This design capitalized on the morphological richness of Chinese VO constructions [e.g., “洗” (xǐ, wash) priming literal “洗衣” (xǐ yī, wash clothes) versus metaphorical “洗脑” (xǐ nǎo, brainwash)] to isolate priming effects. This study extended the Revised Hierarchical Model (RHM; Kroll and Stewart, 1994; Kroll and Tokowicz, 2005; Kroll et al., 2010) and graded embodiment theories and findings (Foroni, 2015; Meteyard et al., 2012; Norman and Peleg, 2022; Reinboth and Farkaš, 2022; Zwaan, 2014) to probe L2 figurative competence.

The results provide support for these hypotheses, revealing a pronounced double dissociation that L2 learners exhibited a reversal (see Table 8): facilitation for literal trials (shorter RTs and lower error rates relative to unrelated baselines) but a marked inhibition for metaphorical trials, primarily reflected in significantly elevated error rates (OR = 2.43, *p* = 0.006, vs. unrelated baselines) alongside a non-significant differences in RTs (*p* = 0.095, *b* = −0.057, vs. unrelated baselines). In contrast, L1 speakers showed uniform dual inhibition (literal and metaphorical, see Table 8), but maintained uniformly low error rates (*M* ± *SD*_L1 Overall_ = 0.07 ± 0.25) and no significant RT differences. This different pattern was confirmed by significant Group × Condition interactions in both RT [*F* (2, 81.36) = 14.27, *p* < 0.001, partial *ω*^2^ = 0.24] and accuracy [*χ*^2^ (2) = 29.99, *p* < 0.001]. The post-hoc contrasts confirmed a large inhibitory magnitude in the error rates of L2 metaphorical conditions (OR = 4.80 vs. literal, *p* < 0.001; OR = 10.20, *p* < 0.001, L2 vs. L1). Individual variability, as captured by strong negative correlations between random slopes (*r* ≈ −0.73 for RT; *r* ≈ −0.97 for errors), underscored the possible role of proficiency and exposure in modulating these effects. This L2-specific reversal is consistent with an embodied conflict account in which strong literal simulations compete with weaker figurative integration, potentially involving increased inhibitory control demands and other possible broader processing costs.

**Table 8:** Priming patterns for L1 and L2 groups in RT and accuracy.

Group	Condition contrast	RT result (*b*, *p*)	ACC result (OR, p)	RT pattern	ACC pattern	Overall pattern
L2 Learners	Literal vs. Unrelated	*b* = −0.138, *p* < 0.001 (significantly faster)	OR = 0.51, *p* = 0.043 (sig. More accurate)	Facilitation	Facilitation	Literal Facilitation (RT + ACC)
	Metaphorical vs. Unrelated	*b* = −0.057, *p* = 0.095 (n.s.)	OR = 2.43, *p* = 0.006 (sig. More errors)	n.s.	Inhibition	Metaphorical Inhibition (ACC only)
	Metaphorical vs. Literal	*b* = 0.082, *p* = 0.001 (significantly slower)	OR = 4.80, *p* < 0.001 (sig. More errors)	Literal Advantage over Metaphorical	Literal Advantage over Metaphorical	Literal Advantage over Metaphorical (RT + ACC)
L1 Natives	Literal vs. Unrelated	*b* = 0.031, *p* = 0.333 (n.s.)	OR = 3.27, *p* = 0.003 (sig. More errors)	Stable	Inhibition	Literal: Stable RT + ACC Inhibition
	Metaphorical vs. Unrelated	*b* = 0.051, *p* = 0.070 (n.s.)	OR = 6.76, *p* < 0.001 (sig. More errors)	Stable	Inhibition	Metaphorical: Stable RT + ACC Inhibition
	Metaphorical vs. Literal	*b* = 0.021, *p* = 0.523 (n.s.)	OR = 2.06, *p* = 0.007 (sig. More errors)	Stable	Literal Advantage over Metaphorical	Stable RT + Literal Advantage over Metaphorical

sig. = significant; n.s. = non-significant. ACC Result reports odds ratios (OR) from the GLMM of error rates: OR < 1 indicates fewer errors (higher accuracy); OR > 1 indicates more errors (lower accuracy).

### 5.2 Interpreting the reversal: dynamic mismatch and increased inhibition control demands

Consistent with classic semantic priming studies that assume uniform facilitation (McNamara, 2005; Meyer and Schvaneveldt, 1971; Neely, 1991), both groups exhibited literal advantages over metaphorical. However, the L2-specific reversal—manifested as inhibition rather than facilitation in metaphorical trials—deviates from these monolingual models and aligns closely with the Declarative/Procedural hypothesis (Ullman, 2001a, 2001b; Ullman and Lovelett, 2018). This hypothesis posits that late-acquired L2 relies more heavily on declarative memory systems, which support explicit and literal representations, whereas L1 processing is predominantly driven by proceduralized routines that enable more automated and efficient handling of both literal and figurative meanings.

From the embodiment cognition perspective, this asymmetry reflects L2-specific embodiment conflicts absent in L1, where unresolved literal simulations hinder flexible abstraction. This pattern closely mirrors the Literal-Salience Resonant Model (Cieślicka, 2006), which argues that literal meanings are obligatorily activated first in L2 idiomatic processing, even in supportive contexts. Eye-movement evidence further corroborates delayed figurative integration and persistent literal salience in L2 (Heredia and Cieślicka, 2016). Such reliance likely stems from decontextualized, instruction-based L2 acquisition, yielding stronger sensorimotor grounding in literal meanings without sufficient experiential attenuation.

Previous studies have shown that Language processing influences sensorimotor systems, producing both facilitatory and inhibitory effects simultaneously (Boulenger et al., 2006; Dudschig et al., 2014; Kaschak and Borreggine, 2008). However, existing research has primarily explained this phenomenon in the context of L1 processing, attributing it potentially to factors such as the stimulus-onset asynchrony (the time interval between stimulus and response, Chersi et al., 2010; García and Ibáñez, 2016). Yet, convincing explanations for this phenomenon in L2 processing remain lacking. More specifically, the observed inhibitory reversal in this study is a situation where L2 learners exhibit priming effects of opposite polarities in literal versus metaphorical conditions, while L1 speakers show uniform inhibition, revealing a language-specific cognitive conflict in L2 metaphorical processing. Juxtaposed against L1 speakers’ relatively stable, efficient processing, L2 metaphorical processing demands a mechanistic explanation that bridges embodied cognition and bilingual models. Drawing on our results—significant Group × Condition interactions in both RT and accuracy, with L2 learners’ pronounced cost in accuracy for metaphorical trials—we interpret these patterns as evidence of a conflict arising from mismatched sensorimotor simulations and abstract semantic integration. This conflict in L2, we argue, potentially involving increased inhibitory control demands and other possible broader processing costs, leading to the behavioral reversal in L2 action metaphor processing.

#### 5.2.1 Source of conflict: dynamic mismatch between literal simulation and metaphorical integration

The observed inhibitory reversal in L2 learners could plausibly arise from general semantic competition or semantic integration difficulty—common in L2 metaphor processing due to weaker lexical-semantic links, lower salience of figurative meanings, or cross-linguistic conceptual mismatch (Chen et al., 2013; Miller et al., 2025). However, language is not a unimodal system of abstract symbols but a multimodal, embodied system in which semantic and conceptual representations are grounded in and constituted by sensorimotor simulations (Zwaan, 2014). In this embodied framework, what appears as domain-general “semantic competition” or “integration difficulty” is in fact a dynamic, multimodal mismatch between strongly activated literal sensorimotor simulations and weaker figurative conceptual mappings.

When processing the shared prime words [e.g., “洗” (xǐ, wash)], both L1 and L2 comprehenders automatically activate sensorimotor simulations grounded in bodily experience (Barsalou, 1999, 2008, 2010; Gibbs, 2012; Gallese and Lakoff, 2005; Glenberg and Kaschak, 2002; Meteyard et al., 2012; Pulvermüller, 2005, 2013; Taylor and Zwaan, 2009; Zwaan et al., 2002; Zwaan, 2014).

In literal trials [e.g.,“洗衣 “(xǐ yī, wash clothes)], this simulation aligns seamlessly with the compositional semantic representation, yielding processing facilitation: shorter RTs and lower error rates relative to unrelated baselines. Our data corroborate this, with L2 learners showing robust literal facilitation (vs. unrelated baseline: *p* < 0.001 in RTs; *p* = 0.043 in errors), suggesting that cross-linguistic bodily experiences (e.g., washing actions) transfer effectively to support basic sensorimotor alignment (Vukovic and Shtyrov, 2014). This facilitation in literal trials aligns with embodied theories positing that basic action language processing shares sensorimotor foundations across languages (Buccino and Mezzadri, 2015).

However, in metaphorical trials [e.g., “洗脑” (xǐ nǎo, brainwash)], the dominant literal simulation (e.g., the action of cleansing with water) clashes with integration of the abstract target domain (e.g., exerting ideological control), creating a potent embodiment conflict.

In L1 speakers, long-term exposure and highly automated mapping of metaphorical concepts optimize this conflict-resolution process through established neural pathways. This efficiency is reflected in minimal behavioral modulation across conditions relative to each other, as evidenced by our data: L1 speakers showed no significant RT differences across conditions (*ps* > 0.070) and uniformly low overall error rates (*M* ± SD = 0.07 ± 0.25), indicating swift and effective conflict resolution that incurs minimal cost at the behavioral level, though related conditions incur task-induced costs relative to unrelated baselines (see below).

In contrast to L1 speakers, L2 learners exhibit greater reliance on declarative memory for metaphorical comprehension, resulting in less stable abstract mappings (Ullman, 2001a, 2001b, Ullman and Lovelett, 2018; Ullman, 2016). The Theory of Experiential Traces (Zwaan and Madden, 2005), within the framework of embodied cognition, posits that bodily experiences play a crucial role in language comprehension, each time we interact with the world, we generate experiential traces similar to those interactions, and subsequently when encountering the same objects or concepts, these traces are activated. Turning to L2 acquisition, explicit, instruction-based learning environments possibly further bias toward the sensorimotor grounding of literal meanings while providing insufficient experiential enrichment to attenuate or re-map these simulations toward abstract domains (Boulenger et al., 2009; De Grauwe et al., 2014; Desai et al., 2011; Pavlenko, 2012; Raposo et al., 2009). Consequently, the strong and persistent literal simulation enters into sustained competition with the fragile abstract mapping, forming the foundation of the “dynamic mismatch” that underlies the observed inhibitory reversal in metaphorical processing.

#### 5.2.2 Increased inhibitory control demands in resolving embodied conflict

This mismatch potentially increased demands for effective inhibitory control to suppress the competing literal simulation while facilitating abstract integration via conceptual mappings (e.g., “Influencing Thought is Cleansing the Mind”). The dynamic mismatch constitutes the intrinsic source of conflict, whereas inhibitory control potentially serves as an important role that influences how this conflict manifests in behavioral outcomes. As proposed by the Inhibitory Control Model (ICM; Green, 1998) and its extensions in bilingual processing (Abutalebi and Green, 2007, 2016; Green and Abutalebi, 2013), achieving the current task goal requires active suppression of interfering representations.

In L1 processing, a mature inhibitory control network—particularly involving the prefrontal cortex, including the dorsolateral prefrontal cortex (DLPFC)—efficiently and precisely suppresses automatically activated but contextually irrelevant representations (Abutalebi and Green, 2007, 2016; Green and Abutalebi, 2013). It further extends to the suppression of competing literal simulations during metaphorical comprehension, allowing seamless abstract integration via conceptual mappings. Converging evidence from neuroimaging and brain stimulation studies supports the causal role of the dorsolateral prefrontal cortex (DLPFC) in this process, particularly in selecting figurative over literal interpretations of idioms and metaphors (Mitchell et al., 2016; Rizzo et al., 2007; Sela et al., 2012; Shibata et al., 2007). This mechanism ensures smooth progression of abstract integration, minimizing conflict costs at the behavioral level. Neuroimaging evidence indicates that the DLPFC plays a key role in domain-general executive control and interference suppression during language processing (Abutalebi et al., 2011; MacDonald et al., 2000), while residual literal salience in conventional metaphors requires baseline inhibitory fine-tuning (Gernsbacher and Faust, 1991; Glucksberg et al., 2001). As a result, even though richer semantic networks are activated, the optimized prefrontal circuitry in L1 speakers rapidly resolves any lingering literal-figurative interference, resulting in swift processing with minimal observable behavioral costs.

In contrast, L2 learners—despite high offline language proficiency (e.g., HSK 5–6 ratings)—face amplified conflict as well as increased inhibitory control demands. Yet the inefficiency of L2 inhibitory control may lead to conflict accumulation during the delayed window in our paradigm and strengthen the interferes, manifesting as the inhibitory reversal: a substantially elevated error rate for metaphorical versus unrelated trial (OR = 2.43, *p* < 0.006) and versus literal trials (OR = 4.80, *p* < 0.001), alongside a non-significant RTs with a trend toward faster RTs (*p* = 0.095), implying the strategic speed-accuracy trade-offs. The random slope correlations in both the RT (*r* ≈ −0.73) and accuracy (*r* ≈ −0.97) models further reveal individual variability in simulation strategies. This metaphorical inhibition is consistent with embodied cognition research showing L2 abstract processing deficits (Boulenger et al., 2009; De Grauwe et al., 2014; Desai et al., 2011; Pavlenko, 2012; Raposo et al., 2009), likely due to reduced immersive experiences in classroom-based, explicit learning environments post-critical period (Chen et al., 2019; Fan et al., 2015). In the context of L2 metaphorical processing, the dominant literal simulation acts as a “non-target” intruder—analogous to L1 lexical interference—and must be actively suppressed to enable successful abstract integration. We argue that for late L2 learners, inhibitory mechanisms remain less practiced, potentially due to reduced bilingual management demands in classroom settings, failing to adequately dampen literal interference within the processing window and thereby giving rise to the observed reversal. This interpretation extends the scope of the Inhibitory Control Model (ICM) beyond code-switching to intra-linguistic resolution of figurative conflicts. The weakness in L2 metaphorical simulation enriches SLA theory by highlighting the role of implicit, experiential grounding over explicit instruction (Ellis, 2002; MacWhinney, 2008).

#### 5.2.3 A different priming pattern: dual inhibition in L1

In addition, although L1 speakers maintained uniformly low overall error rates (*M* ± SD = 0.07 ± 0.25), post-hoc contrasts revealed significant inhibition in both literal and metaphorical conditions relative to unrelated baselines, with metaphorical trials incurring greater costs than literal ones. This pattern of dual inhibition in L1 (literal and metaphorical) likely reflects task-induced semantic relatedness costs commonly observed in sentence processing and plausibility judgment paradigms (e.g., Matsuki et al., 2011; Warren and McConnell, 2007). In such tasks, unrelated pairs (e.g., semantically implausible VO combinations) allow rapid rejection based on minimal semantic integration, whereas related pairs—whether literal or conventional metaphorical—activate richer, more distributed semantic networks in L1 speakers (Barsalou, 1999, 2008; Cree and McRae, 2003), requiring deeper integration and decision processes. Long-term exposure results in denser interconnections among multimodal semantic and sensorimotor nodes (e.g., actions, objects, and abstract mappings for “洗”), triggering broader spreading activation (Collins and Loftus, 1975; Pulvermuller, 1999; Pulvermüller, 2013; Zwaan and Madden, 2005) that may call for additional, albeit modest, suppression of momentarily irrelevant features. The delayed-response window in our paradigm may further amplify these costs by allowing additional time for uncertainty accumulation during evidence evaluation (like noisy-channel models of rational comprehension; Gibson et al., 2013). Notably, even conventional metaphors retain residual literal salience in monolinguals (Desai et al., 2011), which can contribute to modest decision costs through routine, automatic suppression processes during comprehension.

To conclude, the polarity reversal in L2 learners (literal facilitation vs. metaphorical inhibition, primarily evident in error rates) contrasts qualitatively with the dual inhibition (literal and metaphorical inhibition) observed in L1 speakers, who nevertheless achieved stable and efficient resolution of the inhibition. This L2-specific dissociation underscores the embodiment conflict central to our hypotheses. Priming thus functions as “double-edged sword”—facilitative in convergence but suppressive in conflict. For L2 learners, the inhibitory reversal was most pronounced in error rates for metaphorical trials (OR = 2.43, *p* = 0.006 vs. unrelated), with no significant RT difference (*b* = −0.057, *p* = 0.095, vs. unrelated). This dissociation indicates that accuracy is a more sensitive measure of the underlying embodied conflict in L2 processing, and that L2 learners may adopt a strategic speed-accuracy trade-off, prioritizing rapid responses amid unresolved conflict at the expense of decision quality.

Furthermore, we acknowledge that several alternative explanations could also contribute to the priming facilitation turning into inhibition, thereby driving the observed “reversal,” including: (1) conventionalized metaphors are more likely to possess L1–L2 conceptual mismatches compared with literals; (2) potential speed-accuracy trade-offs under the delayed-response design, primarily evident in accuracy rather than RTs as shown; (3) strategic differences likely induced by the isolated response window. Nevertheless, the specific morpheme-mediated priming paradigm used in this study—particularly the use of a shared free verb morpheme [e.g., “吃” priming “吃亏” (chī kuī, literally “eat loss,” figuratively “suffer a loss”)] to pre-activate a strong literal sensorimotor simulation before the target VO construction—allows us to isolate and directly test the embodied conflict between automatic literal action simulation and fragile figurative integration. This design feature provides compelling evidence that the dynamic embodied mismatch lies at the core of the observed priming effects in opposing directions in L2 learners, offering an explanation that goes beyond domain-general semantic competition and accounts for both literal and metaphorical language processing.

### 5.3 Implications

Our findings, revealing a polarity reversal in semantic priming for conventional action metaphors in L2 Chinese learners, carry implications for theories of embodied cognition, bilingual processing models, and second language acquisition (SLA), with practical applications for SLA and the teaching of figurative language.

#### 5.3.1 Theoretical implications

First, this study extends the theories of embodied cognition. Research on embodied cognition in L2 has often centered on the fundamental question of its mere existence (i.e., “whether it is embodied”). Our findings provide behavioral evidence for “graded embodiment” (Zwaan, 2014), demonstrating strong literal sensorimotor simulations and attenuated or absent figurative ones. More importantly, this study shifts the focus toward the conditional nature of embodiment—specifically, how the content and strength of a simulation determine its real-time fit with target semantics. For instance, in processing culture-bound metaphors like “wash brain,” the literal simulation cued by the verb stem conflicts with the required abstract mapping, thereby obstructing semantic access. Consequently, a critical direction for future research is to investigate how the intensity of a given simulation modulates inhibitory demands, and how the cognitive system dynamically regulates these multimodal activations during comprehension.

Second, the “reversal” effect documented in this study offers a dynamic new perspective for understanding differences in conceptual access between a first and second language. This study holds that L2 metaphorical processing involves heightened conflict resolution demands, which may recruit inhibitory control processes, although the present behavioral data do not allow us to isolate the specific contribution of inhibitory control from other mechanisms such as semantic integration difficulty or L1–L2 conceptual mismatch.

It offers a more refined account of the shallow-structure hypothesis (SSH; Clahsen and Felser, 2006), which holds that L2 processing relies heavily on surface cues. In the present study, the advantage observed for literal trials stems precisely from such surface dependence: the shared verb morpheme activates robust sensorimotor simulations that support comprehension. Yet the same surface cue provokes conflict when the item is metaphorical. This demonstrates that “reliance on surface form” is a double-edged sword: when the simulation it triggers aligns with the intended meaning, processing is facilitated; when it clashes, and deeper integrative mechanisms are still fragile, the conflict generates disproportionate interference. Thus, L2 “shallowness” may not be a global characteristic, but rather a selective signature that emerges whenever form–meaning mappings become opaque or cross-modal competition arises.

It also offers a fresh lens on how L1 and L2 differ in conceptual access. The classic Revised Hierarchical Model (RHM; Kroll and Stewart, 1994) stresses a fixed imbalances in lexical-to-conceptual links; our results show that, even at the conceptual level, the two languages can diverge in both simulation strength and integration efficiency. L2 learners may command the declarative knowledge of metaphors such as “brainwash,” yet fail to automatize the integration of sensorimotor simulations and the abstract conceptual domain. This gap is rooted in distinct learning trajectories: L1 concepts emerge within vivid, multimodal immersion, whereas adult L2 is often acquired through explicit, decontextualized methods (word lists, translation, etc.). Thus, bilingual differences hinge not only on connection weights but also on the tightness of cross-modal binding and the real-time resolution of such bindings. The “reversal” effect is a signature of this adaptive bilingual cognition.

The findings provide clear evidence for inhibitory-control models in within-language processing tasks. Mainly linked to cross-language conflict (e.g., code-switching; Green, 1998), inhibition here proves critical when L2 learners handle literal-metaphorical rivalry in L2. The elevated error rate observed in L2 during metaphor processing signals struggles in suppressing a dominant yet off-target embodied simulations for a subtler abstraction paths. Thus, inhibitory control is stretched from broad language choices to fine-grained semantic and modal picks.

Third, this study reveals the fundamental challenge in late second language conceptual acquisition.

It broaden the “declarative/procedural model” (Ullman, 2001a, 2001b). The reversal shows that even advanced learners who can verbalize what a metaphor means still fail to automate its on-line integration. Unlike L1 speakers, they cannot unconsciously coordinate—or suppress—literal sensorimotor simulations triggered by the action verb and therefore cannot access the abstract meaning swiftly. This “know-do” indicates that declarative memory can store the rule, yet the procedural system that should turn that knowledge into fluent, real-time blending remains under-developed. The reversal marks this unfinished proceduralization behaviorally.

It also illuminates how “input and acquisition mode” shape conceptual representations. L1 concepts emerge naturally from rich, multimodal, socially interactive immersion. In contrast, adult L2 concepts are usually acquired after the critical period and often originate from decontextualized, explicit instructional methods (e.g., vocabulary lists, translation, component analysis). This can lead to “flattened” and “decontextualized” L2 representations. Taking “brainwash” as an example, its representation may be reduced to a vague label loosely linked to the isolated action verb “wash,” lacking the interwoven event structure, affective dimensions, and cultural scripts inherent in L1 representations. These representational features are the very source of simulation conflict and processing difficulty, suggesting that the depth (experiential richness) and mode (implicit/explicit) of input are stronger predictors of deep conceptual competence than input quantity alone.

#### 5.3.2 Practical implications

The empirical evidence from this study directly informs pedagogical strategies for teaching conventional VO metaphors in CSL. A challenge identified is the pedagogical tendency to inadvertently reinforce the very “simulation mismatch” that hampers comprehension. Traditional instruction often begins with a decontextualized decomposition of metaphors—for example, presenting “洗脑” (xǐ nǎo, brainwash) as the sum of “wash” + “brain.” While intended to clarify, this approach risks cementing a strong literal sensorimotor simulation (the physical act of washing) that actively competes with the target abstract meaning during online processing.

Therefore, the core pedagogical principle must shift decisively from deconstructing literal components to constructing holistic metaphorical events. The goal is to bypass the entrenched literal simulation path and guide learners in directly building a coherent, multimodal “event simulation” that embodies the metaphor’s figurative meaning. For “brainwash,” this means moving away from any association with cleaning and instead simulating the event of “systematic ideological persuasion and thought reform”. This can be operationalized through structured, experiential tasks. For instance, to embody the core feature of “information control”—a prerequisite for brainwashing—learners can engage in a sensory restriction exercise: closing their eyes, using noise-canceling headphones, or limiting physical movement. This immediate, bodily experience of “isolation” serves as a direct, non-linguistic anchor for the abstract concept. Subsequently, this embodied understanding should be elaborated through richer, multi-modal contexts. Techniques such as role-playing (e.g., simulating a scenario of coercive persuasion), creating situational scripts, or employing virtual reality can immerse learners in a fuller social context where the metaphor is used. Follow-up tasks requiring learners to explain or represent the metaphor through gesture, sketching, or novel figurative language force the integration of multimodal resources, consolidating the “holistic event simulation” and weakening dependence on the literal interpretation.

### 5.4 Limitations and future directions

Despite these contributions, several limitations of the present study should be noted. First, although the final sample size (*N* = 90 after exclusions) was sufficient to detect medium effect sizes (*f* ≈ 0.183), the participant groups lacked sufficient diversity, limiting the generalizability of the findings. Specifically, the L2 learners were restricted to those at HSK Levels 5–6, without further subgrouping (e.g., lower proficiency levels such as HSK Level 1–3 or higher levels such as HSK 7–8), which may mean that the observed patterns do not extend to less or more proficient learners. Additionally, both groups were predominantly female, potentially introducing bias, as females may exhibit slightly stronger inhibitory control in language tasks. The age difference between groups was only marginally significant, with L2 learners tending to be slightly older; this warrants further refinement in future work. Moreover, the L2 participants had 1–5 years of immersion in China, leaving unclear how longer exposure or immersion duration might modulate the observed asymmetry (literal facilitation vs. metaphorical inhibition). This pattern may represent a transitional phase, but longitudinal tracking is further needed to test the gradient predictions. To address these issues, future studies should recruit more diverse samples, encompassing a broader range of proficiency levels, and incorporate immersion experience as a covariate to partition variance. This would enhance the applicability of the conclusions to a wider array of L2 learners.

Second, reliance on behavioral measures alone, while ecologically valid, offers limited insight into the neural substrates underlying the reversal effect. Error-based reversals may stem from downstream integration failures, but their temporal dynamics—such as simulation onset (N400) or inhibitory processes (LPC)—can only be inferred indirectly as potential accounts. Although the delayed-response paradigm effectively isolated embodied dynamics from motor artifacts, neuroimaging is required to clarify whether metaphorical inhibition reflects sustained literal motor resonance (mu suppression), heightened semantic competition (amplified N400), or elevated executive demands (late frontal negativity). Future replications could integrate concurrent EEG-fMRI or MEG to provide multimodal validation and localize the reversal within sensorimotor, semantic, or control networks.

Third, the delayed-response paradigm employed a relatively long 4,000-ms response window that may amplify strategic or metacognitive processes during the extended interval. However, several features of the design minimize this concern: (1) the plausibility judgment task combined with explicit instructions to respond as quickly and accurately as possible once the prompt appeared encouraged rapid, online decision-making; (2) the morpheme-mediated priming design itself generated a strong, automatic embodied conflict (strong literal simulation clashing with weak figurative integration) that is difficult to override through strategic re-analysis; (3) the observed pattern—facilitation in literal trials but clear inhibition (elevated errors) in metaphorical trials—aligns with predictions of automatic embodied competition rather than deliberate, controlled processing. Nevertheless, future studies could systematically compare immediate versus delayed versions of the paradigm to further isolate automatic from controlled contributions and strengthen causal claims about sustained literal simulation conflicting with L2 metaphorical integration.

Fourth, the Vietnamese–Chinese bilingual comparison, while novel in its non-Indo-European focus, may overemphasize literal–action overlaps (e.g., shared ‘ingestion’ schemas) while underestimating typological differences; in alphabetic L2s, morphological priming might weaken, potentially diminishing reversals. Furthermore, Sino-Vietnamese word overlaps (i.e., vocabulary in Vietnamese derived from Chinese origins) could enhance literal transfer. Future research should contrast unrelated L1s (e.g., English speakers) for validation.

## 6 Conclusion

In summary, this study documented a clear “reversal” (literal facilitation vs. metaphorical inhibition, primarily evident in error rates) in the role of priming among L2 Chinese learners in an action-verb-morpheme-mediated priming task. These findings clarify one of the L2 asymmetries: dynamic mismatches between sensorimotor simulations and semantic integration, involving increased inhibitory control demands and other possible broader processing costs. Beneath this reversal lies a shift in L2 processing strategies: L2 learners potentially display cautious, effortful adaptations (e.g., speed-accuracy trade-offs) to resolve embodied conflicts, whereas L1 speakers potentially display efficient, automated adjustments of cognitive resources, manifesting as stable RTs and overall low error rates despite a dual inhibition (literal and metaphorical). This pattern highlights a key bottleneck in L2 semantic competence and provides a fresh interpretive lens for difficulties encountered in naturalistic settings. At its core, attaining L1-like L2 proficiency depends on developing a flexible, precise semantic system capable of effectively managing embodied resources. In particular, enriching the experiential basis of perceptual-motor simulations may enable L2 learners to progress toward L1-like efficiency in processing. Future work might incorporate neuroimaging techniques (e.g., EEG, fMRI) or gesture-enhanced interventions to examine the causal contributions of perceptual-motor enrichment in mitigating L2 embodied conflicts and supporting this shift from qualitative to quantitative processing, thereby shedding further light on the neural plasticity underlying cross-linguistic embodied simulation.

## Data Availability

The raw data supporting the conclusions of this article will be made available by the authors, without undue reservation.

## Footnotes

1 95% CIs for *ω*_*p*^2^ is one-sided, upper bound truncated at 1.00. *ω*_*p*^2^ is theoretically bounded between 0 and 1, and its 95% CI was computed using the noncentral F distribution (*effectsize* package; Ben-Shachar et al., 2020). When the estimated upper bound exceeds the theoretical maximum of 1, it is truncated to 1.00—a standard handling of mathematical parameter boundaries. Thus, the 95% CI [0.02, 1.00] indicates the true *ω*_p^2^ is at most 1, with the lower bound providing the more informative estimate.

## Supplementary material

Supplementary Table S1 Representative critical stimuli.

Supplementary Table 2 Exact sum-to-zero contrast matrices.
