# Supplementary material for: Inhibitory reversal of morpheme-mediated semantic priming in L2 Chinese: embodiment conflicts in conventional action metaphor processing
Source: Front Psychol. 2026 Mar 31;17:1778678. doi: 10.3389/fpsyg.2026.1778678 (PMC13076550; doi:10.3389/fpsyg.2026.1778678)
Supplement: Supplementary Table 2 — Exact sum-to-zero contrast matrices. [file Table_2.DOCX]

### Supplementary Table S2

**Exact sum-to-zero contrast matrices used in the models**

**Group factor** (2 levels: L1 Chinese speakers, L2 learners)

| **Level** | **Contrast1** |
| --- | --- |
| L1 Chinese speakers | +1.0 |
| L2 learners | -1.0 |

**Condition factor** (3 levels: unrelated, metaphorical, literal)

| **Level** | **Contrast1** | **Contrast2** |
| --- | --- | --- |
| Unrelated | +1.0 | 0.0 |
| Metaphorical | 0.0 | +1.0 |

**Note.** These contrast matrices were automatically generated by R’s contr.sum() function based on the explicit factor level order defined: L1 speakers of Group and the unrelated condition of Condition are set as the respective base levels for matrix construction. The same coding scheme was applied to both the reaction time (LMM) and accuracy (GLMM) models.

- **Group Contrast1**: Represents the deviation of L1 Chinese speakers from the grand mean (positive coefficient indicates L1 above grand mean; L2 deviation = −coefficient). The full group difference (L2 − L1) = 2 × |coefficient|.
- **Condition Contrast1**: Unrelated vs. average of metaphorical and literal (negative coefficient indicates fewer errors / shorter RTs in the average of metaphorical and literal relative to unrelated).
- **Condition Contrast2**: Metaphorical vs. literal (positive coefficient indicates more errors / longer RTs for metaphorical relative to literal; unrelated has zero weight).

In sum-to-zero coding, the model intercept equals the grand mean (overall average across all levels). Each contrast coefficient represents the corresponding deviation from this grand mean. The literal level deviation is derived as −(Contrast1 coefficient + Contrast2 coefficient).

This supplementary table is referenced in the Method (Section 3.5.2 Statistical Analysis) and Results sections (Tables 4 and 6 footnotes) to ensure full transparency of the coding scheme.
